# Supplementary material for: Simulated operant reflex conditioning environment reveals effects of feedback parameters
Source: PLoS One. 2024 Mar 21;19(3):e0300338. doi: 10.1371/journal.pone.0300338 (PMC10956789; doi:10.1371/journal.pone.0300338)
Supplement: S1 Table — Values represent overall mean ± SE. Tukey HSD was used for pair-wise comparisons (↔) between conditions of high and low variability (HV, LV) and different feedback types (Kp, Kr, KpKr). Statistical significance (* p<0.05, **p<0.01, ***p<0.001, ****p<0.0001). (PDF) [file pone.0300338.s001.pdf]

Supporting Information

S1 Table. Effect of biological variability and feedback type on performance and operant strategy (moderate threshold). Values represent overall mean ± SE. Tukey HSD was used for pair-wise comparisons (↔) between conditions of high and low variability (***HV, LV***) and different feedback types (***Kp, Kr, KpKr***).

Statistical significance (* $p<0.05$, **$p<0.01$, ***$p<0.001$, ****$p<0.0001$)

	**Performance**		**Strategy**	
**Variability / Feedback**	***LV***	***HV***	***LV***	***HV***
Kp	0.677±0.006	0.796±0.006	14.847±0.977	10.038±0.882
Kr	0.658±0.006	0.784±0.006	10.052±0.959	11.808±1.137
KpKr	0.652±0.005	0.746±0.008	11.898±1.283	10.651±1.563
Kp ↔ Kr	0.019±0.0011(*)	0.012±0.001(ns)	4.795±0.093(****)	1.771±0.090(ns)
Kr ↔ KpKr	0.028±0.001(ns)	0.048±0.001(****)	2.502±0.130(ns)	1.389±0.165(ns)
Kp ↔ KpKr	0.011±0.001(**)	0.038±0.002(****)	2.324±0.152(ns)	0.063±0.184(ns)
