# Supplementary material for: Simulated operant reflex conditioning environment reveals effects of feedback parameters
Source: PLoS One. 2024 Mar 21;19(3):e0300338. doi: 10.1371/journal.pone.0300338 (PMC10956789; doi:10.1371/journal.pone.0300338)
Supplement: S2 Table — Values represent overall mean ± SE. Tukey HSD was used for pair-wise comparisons (↔) between conditions of high and low variability (HV, LV) and difficult, moderate, and easy threshold (DT, MT, ET). Statistical significance (* p<0.05, **p<0.01, ***p<0.001, ****p<0.0001). (PDF) [file pone.0300338.s002.pdf]

S2 Table. Effect of biological variability and reward threshold on performance and operant strategy. Values represent overall mean ± SE. Tukey HSD was used for pair-wise comparisons (↔) between conditions of high and low variability (***HV, LV***) and difficult, moderate, and easy threshold (***DT, MT, ET***).

Statistical significance (* p<0.05, **p<0.01, ***p<0.001, ****p<0.0001)

	**Performance**		**Strategy**	
**Variability / Feedback**	***LV***	***HV***	***LV***	***HV***
LT	0.661±0.004	0.784±0.007	18.753±1.297	13.378±1.166
MT	0.658±0.006	0.784±0.006	10.052±0.959	11.808±1.137
HT	0.729±0.005	0.797±0.006	6.364±1.317	8.252±1.224
LT ↔ MT	0.004±0.001(ns)	0.002±0.001(ns)	8.905±0.146(****)	1.656±0.146(ns)
MT ↔ HT	0.069±0.001(****)	0.013±0.001(ns)	3.619±0.194(**)	3.351±0.177(*)
LT ↔ HT	0.066±0.009(****)	0.015±0.001(ns)	12.476±0.127(****)	5.069±0.085(**)
