# Supplementary material for: Simulated operant reflex conditioning environment reveals effects of feedback parameters
Source: PLoS One. 2024 Mar 21;19(3):e0300338. doi: 10.1371/journal.pone.0300338 (PMC10956789; doi:10.1371/journal.pone.0300338)
Supplement: Attachment — Submitted filename: Response to Reviewers_v2.docx [file pone.0300338.s003.docx]

We thank the editor and reviewers for their valuable comments that have strengthened the manuscript. We have carefully reviewed these comments and we believe they have been sufficiently addressed. We have primarily addressed the comments in the manuscript but have included excerpts in this document where we felt necessary. Our responses are in red font.
The line numbers refer to lines in ‘Track Changes (Simple Markup)’ mode. Thank you very much for your time, effort, and attention.

Journal requirements:

1. Please ensure that your manuscript meets PLOS ONE's style requirements, including those for file naming.

>> Thank you for the clarification of the guidelines. We have thoroughly reviewed PLOS ONE style template guidelines and have made major changes in our manuscript.

2. We note that the grant information you provided in the ‘Funding Information’ and ‘Financial Disclosure’ sections do not match.

>> Thank you for the comment. Please indicate what was inconsistent.

3. Please update your submission to use the PLOS LaTeX template. The template and more information on our requirements for LaTeX submissions can be found at <http://journals.plos.org/plosone/s/latex>.

>> This is little bit confusing. We were asked to submit it as the doc file. Is there a reason to convert it to LaTeX?

4. We note that you have stated that you will provide repository information for your data at acceptance. Should your manuscript be accepted for publication, we will hold it until you provide the relevant accession numbers or DOIs necessary to access your data. If you wish to make changes to your Data Availability statement, please describe these changes in your cover letter and we will update your Data Availability statement to reflect the information you provide.

>> Thank you for the comment. We included the appropriate URL.

5. Please include your full ethics statement in the ‘Methods’ section of your manuscript file. In your statement, please include the full name of the IRB or ethics committee who approved or waived your study, as well as whether or not you obtained informed written or verbal consent. If consent was waived for your study, please include this information in your statement as well.

>> Thank you for the clarification. We have included the full ethics statement in the Methods section. This study was considered as exempt submission, but we have collected verbal consent from each participant prior to their participation. (Lines 128-130)

The study was considered exempt by the University of Texas at Austin Institutional Review Board. While consent was not required, it was acquired via email or verbally from all participants prior to participation.

6. We note that Figures 1 and 3 in your submission contain copyrighted images.

>> Thank you for the comment. We created Figures 1 and 3, so we are not sure how this violates any copyright. Could you please be more specific on how these are copyrighted?

Reviewer Comments:

Reviewer 1

1. The study presents the results of original research.
However, there are several studies that have looked at these types of tasks and the strategy used across learner types within both implicit and explicit domains. It would improve the scholarship and emphasis on originality of this research by including the following references.

>> Thank you for the suggested references. We have added a suggested reference (Holland et al., 2018) in our discussion section to address the importance of exploring the effect of reward on subject’s strategy during reward-based learning.

Lines 458-60:

Previous work in visuomotor learning [39] has emphasized the effect of negative feedback (e.g., failure) on increased variability of subject’s motor strategy during reward-based learning.

2. Results reported have not been published elsewhere.
I believe this to be correct, with the exception of a preprint in bioRxiv.

>> Correct.

3. Experiments, statistics, and other analyses are performed to a high technical standard and are described in sufficient detail.

I am uncertain of how the learning of this task is equivalent to learning how to modulate actual H-reflex. Is there prior evidence that shows how learning capability of this task is related to individual capability to change H-reflex?

>> Thank you for the valuable comment. We have clarified this in our discussion section (Lines 497-99). To the best of our knowledge, there has not been any published literature that has investigated the role of feedback parameters on explicit aspects of learning during the operant H-reflex conditioning process. In the introduction we have stated this is a novel study.

Referencing to our previous simulation work that focused on fMRI neurofeedback (Oblak et al., *PLOS Comp Bio* 2017) and with assumption of unimpeded perception, we hypothesized that we could isolate the effects of simulated feedback on explicit decision-making processes likely occurring during actual neural operant conditioning. The goal of this study was to suggest a novel approach in understanding the role of feedback parameters in tasks that are similar to neural operant conditioning. However, further investigation is required to show direct connection of how learning of this simulation task can be transferred to real operant H-reflex conditioning capability.

Lines 497-99

Our simulation paradigm possesses two major limitations that preclude drawing a direct inference of how learning from simulation may be transferred to real operant H-reflex conditioning capability.

Clarification on how learning of this task is related to learning of H-reflex is needed. What is lost if the simulated H-reflex is removed and every step after decision gain in figure 1 is maintained. What difference in visual feedback would this create that requires the simulated H-reflex to be included?

>> Thank you for the valuable comment. We have clarified the role and reason behind adding the biological variability and signal noise, when generating the simulated H-reflex (e.g., hsim).

Lines 105-09:

As the inherently variable nature of H-reflex amplitude has been shown to be correlated with the fluctuations in motoneuronal membrane potential [27], application of data-driven biological variability and signal noise enabled our simulation environment to be as similar to the neurophysiological environment of operant H-reflex conditioning.

I do recognize that you compare the virtual performance here to that of real performance of H-reflex conditioning from previously collected data [23]. However, this would at best represent an association between these types of learning and not necessarily a transfer of learning.

>> Thank you for pointing out this oversight. It is correct that the goal of this study is to represent the association between the types of learning during the 1) real-data driven simulation task, and 2) real operant H-reflex conditioning. We have made it clear by adding the statement.

Lines 116-18:

We then compared this model to real operant H-reflex conditioning performance to demonstrate the similarity in learning processes between the simulation environment and that of operant H-reflex conditioning.

What is the y axis of figure 7?

>> Thank you for pointing out this mistake. We have added the axis label for the Figure 7 as below.

In the mixed effect model did you also include trial number as a fixed effect?

>> Thank you for this helpful idea. Previously we tested additional models that included “run number” as an additional fixed effect. However, our analysis has shown no significant difference between the models.

Is the outcome variable for the trained LMM the same as hsim? Do you train an LMM on the virtual reflex data and then test it on the real data?

>> Thank you for this valuable question. We have clarified how we generated LMM in our methods section.

Lines 292-95:

Using this LMM driven from the simulated environment, we then input measured (i.e., real) H-reflex variability extracted from data collected previously for five healthy and two participants post-stroke performing operant RF H-reflex conditioning [23], to calculate the estimated performance.

I am having difficulty translating your stat method to figure 7. I thought hsim ranged from 0 to 1 but figure 7 shows an outcome variable ranging from 50 to 250?

>> Thank you for your comment and pointing this out. We have made clarification of hsim range in Figure 2. Also, we have added a sentence explaining the transition from raw hsim magnitude to normalized value (Lines 295-97).

Lines 295-97:

Later, we normalized the estimated performance magnitude for direct comparison with real operant H-reflex conditioning performance, using the method mentioned in our previous study [23].

Also, for the fixed effect of variability, is that the biological variability or some other form of variability?

>> Thank you for pointing out this oversight. I have clarified the term “variability” as “biological variability”.

Line 290-292

We included the performance mean across a single run as the dependent variable with the fixed effect of biological variability and the random effect of participant.

Typically, in validation, although correlation is acceptable, other metrics of validation are MAE, MSE and RMSE. Additionally, you may consider replacing figure 7 with a Bland-Altman plot to demonstrate the agreement between real and simulated. Lastly, I think you need to provide some threshold by which the model is validated or not. To have a strong correlation in one out of 7 test participants isn’t strong evidence of validation. Alternatively, you could generate random data to be compared to the validated data and perform a contrast between the error in predictive accuracy.

>> Thank you for this valuable insight. After careful consideration, we should not have used the word “validation”. The purpose of comparison was to show the similarity of learning processes. We made the following changes.

Lines 116-18:

We then compared this model to real operant H-reflex conditioning performance to demonstrate the similarity in learning processes between the simulation environment and that of operant H-reflex conditioning.

Lines 285-88:

### Similarity of learning process during simulated environment: Comparison with real Operant H-reflex conditioning

To illustrate similarities between learning in the simulated environment and actual operant H-reflex conditioning behavior, we used a statistical model.

Please provide a little more context for how to interpret hsim, the primary performance outcome measure. Please provide an example of what an hsim of 1 versus an hsim of 0 represents.

>> Thank you for your comment. I have clarified the interpretation of hsim magnitudes.

Lines 211-13:

In other words, having a peak-to-peak value closer to 0.5 implied the subject was successful in matching the hidden target, which can be translated as success in the context of down-conditioning, whereas value closer to 1 implied failure.

Could you please provide a visual of the Kr versus Kp versus KpKr display that participants would see under each condition?

>> Thank you for your comment. We have previously added the difference in visual feedback between Kr versus Kp versus KpKr in Figure 1. We have also added reference of the figure in the methods section as below.

Lines 230-44:

During ***Kp***, a blue bar was presented (**Fig 1B, top right**), in which the height was peak-to-peak magnitude of the simulated H-reflex. No bar color change was associated with ***Kp*** feedback. For ***Kr***, the bar color changed based on the result of the comparison between the simulated H-reflex magnitude and the pre-determined threshold levels (**Fig 1B, middle right**). The threshold levels were chosen as the 44th, 66th, and 77th percentile of the 1,350 evoked H-reflexes without feedback [23], designated as easy/moderate/difficult thresholds (***ET***=0.46, ***MT***=0.7 and ***DT***=0.96, respectively). If the magnitude of the simulated H-reflex was below the threshold, the bar turned green indicating success and the cumulative score increased. If $h_{sim}\left( k \right)$ was larger than the threshold, the bar turned red, indicating failure and the score decreased. The bar height and the threshold were kept hidden from the participants during the ***Kr*** condition and only the binary result with fixed bar height was provided as feedback. Not introduced in the Demonstration, but used later in the Experiment, is the combination of ***Kp*** and ***Kr*** feedback, which is the ***KpKr*** condition, but with a moving bar height as described above (**Fig 1B, bottom right**), reflecting current operant H-reflex conditioning practices [14].

Can you please include individual dots in the bar graphs to represent individual participant performance. It would also be good to use boxplots instead of bar graphs.

>> Thank you for your valuable suggestion. We have replaced the bar graph plot to boxplots as suggested and have added scatter dot plot to convey more information, as shown in Figures 5 and 6:

You provide pairwise comparisons but condition performance should also be reported.

>> Thank you for your comments. To show mean and standard error value of each condition’s performance and strategy across conditions, we have added Supplemental Information table (S1 Table 1. and 2.).

In the results please provide model estimates and confidence intervals of performance across conditions and interactions.

>> Thank you for this valuable suggestion. Our preference is to use mean difference and standard error (SE) for pair-wise comparisons as they can quickly provide an intuitive sense of the differences between conditions, however we are open to modifying if there is a specific advantage to this.

Although you demonstrate that mean group performance among the LV/KP condition had the worse performance and was the most aggressive this doesn’t really tell us if greater aggression/exploration is related to worse performance or if this is the case across all conditions. A similar situation appears in the reward threshold to variability result as well. IT would be helpful to see scatter plots of aggression versus performance stratified by group to better understand if these data support hypothesis 3: “the difficult reward criterion will worsen performance and make operant strategy less aggressive”.

>> This is a fascinating question and part of our plan for future analysis of the data. To sufficiently answer this question on the relation between aggressiveness and performance, our future work based on these data will be to determine how these relate on a trial-by-trial basis. To the reviewer’s point, we did look at any subject-based correlations and did not find any significant relations (see example below). Other conditions did not show any correlations either. Thus, our opinion is that a trial-by-trial basis analysis, which is out of the scope of the current paper, would be best to investigate this question.

4. Conclusions are presented in an appropriate fashion and are supported by the data.
I think the conclusions are presented well but I am not sure that results from this study indicate that explicit processes play a role in operant H-reflex conditioning, and this process is modulated by feedback parameters. I think the results the role of explicit processes on a visuomotor skill and how they are modified due to feedback parameters and feedback noise.

>> Thank you for your comment. We made changes to the first paragraph of the discussion and the conclusion sections to soften the certainty of relation between two paradigms.

Lines 411-27:

The goal of the present study was to investigate the effect of feedback type, biological variability, and reward threshold on individuals’ feedback performances and operant strategies in a simulated operant H-reflex conditioning environment. We used a novel simulation environment based on real operant H-reflex conditioning parameters to isolate the effect of feedback parameters on explicit learning. Our main findings, consistent with our hypotheses, were that 1) Kr feedback resulted in better performance than Kp feedback alone, 2) larger biological variability worsened feedback performance, 3) biological variability modulated the effect of feedback type on operant strategy, 4) biological variability modulated the effect of reward threshold on strategy, and 5) a difficult reward threshold resulted in better performance and more aggressive operant strategies. Performance in the simulated environment, albeit governed entirely by explicit learning processes, was found to be similar to actual operant H-reflex conditioning performance. This study is a new approach to understanding the learning mechanisms of operant H-reflex conditioning. Our results suggest that explicit processes play a role in operant H-reflex conditioning, and this process is modulated by feedback parameters. This paradigm can be used to quickly examine different feedback parameters on operant learning and potentially identify non-responders, ultimately improving the procedural robustness of self-modulation of H-reflex activity.

Lines 512-24:

We developed a simulated environment of operant H-reflex conditioning to investigate the effects of feedback parameters on explicit learning. The model explained a large portion of the variance of the real H-reflex conditioning despite lacking any implicit learning process. This simulated paradigm may potentially allow the investigation of parameters beyond the capability of real operant H-reflex conditioning and far more efficiently. Our model suggests that the conditions for best operant H-reflex conditioning performance is to provide both reward and error feedback with at least a moderate (66th percentile success rate) threshold, particularly with low biological variability of the H-reflex. Operant strategy is most aggressive at low variability with error feedback (knowledge of performance) and with a more difficult reward threshold. As variance increases, the effects of feedback type and reward threshold are not as strong. The future of this simulation paradigm may provide a better understanding of learning strategies and ability to identify non-responders, ultimately enhancing the robustness of operant H-reflex conditioning.

Reviewer 2

1. The introduction lacks a clear explanation of the significance of the research and its potential applications.

>> Thank you for your valuable suggestion. I have clarified the significance and potential applications of this study.

Lines 118-23:

This experiment represents a novel approach to understanding the role of feedback parameters in explicit learning that is likely taking place during neural operant conditioning. The simulated operant H-reflex conditioning environment provides an efficient method for analyzing and possibly improving learning. This approach may also assist in identifying non-responders, ultimately enhancing the robustness of operant H-reflex conditioning.

2. While the research aims to determine the explicit decision-making processes in response to feedback, the specific research questions or hypotheses are not explicitly stated in the introduction. It would be useful to outline the research objectives clearly.

>> Thank you for the pointing out this oversight. We have clarified our research hypotheses in the introduction section.

Lines 97-100:

Our hypothesis was that several factors likely affected explicit learning: 1) ***Kr*** feedback will improve performance compared to ***Kp*** feedback [26], 2) larger biological variability will result in worse performance, and 3) the reward threshold modulates performance and strategy.

3. The manuscript briefly describes the experimental setup but lacks sufficient detail about the web application game and the operant conditioning model. A more comprehensive description of the methodology, including the design of the game and how feedback was provided, would enhance the paper's clarity.

>> Thank you for this insightful comment. We have made major changes for clarification of the experimental setup in our method section.

Lines 211-213:

In other words, having peak-to-peak value closer to 0.5 implied the subject was successful in matching the hidden target, which can be translated as success in the context of down-conditioning, whereas value closer to 1 implied failure.

Lines 230-244:

During ***Kp***, a blue bar was presented (**Fig 1B, top right**), in which the height was peak-to-peak magnitude of the simulated H-reflex. No bar color change was associated with ***Kp*** feedback. For ***Kr***, the bar color changed based on the result of the comparison between the simulated H-reflex magnitude and the pre-determined threshold levels (**Fig 1B, middle right**). The threshold levels were chosen as the 44th, 66th, and 77th percentile of the 1,350 evoked H-reflexes without feedback [23], designated as easy/moderate/difficult thresholds (***ET***=0.46, ***MT***=0.7 and ***DT***=0.96, respectively). If the magnitude of the simulated H-reflex was below the threshold, the bar turned green indicating success and the cumulative score increased. If $h_{sim}\left( k \right)$ was larger than the threshold, the bar turned red, indicating failure and the score decreased. The bar height and the threshold were kept hidden from the participants during the ***Kr*** condition and only the binary result with fixed bar height was provided as feedback. Not introduced in the Demonstration, but used later in the Experiment, is the combination of ***Kp*** and ***Kr*** feedback, which is the ***KpKr*** condition, but with a moving bar height as described above (**Fig 1B, bottom right**), reflecting current operant H-reflex conditioning practices [14].

Lines 287-299:

To illustrate similarities between learning in the simulated environment and actual operant H-reflex conditioning behavior, we used a statistical model. We computed a single linear mixed model (LMM) based on conditions C9 and C10 from all participants in the simulated environment (***KpKrLVMT*** and ***KpKrHVMT***). We included the performance mean across a single run as the dependent variable with the fixed effect of biological variability and the random effect of participant. Using this LMM driven from the simulated environment, we then input measured (i.e., real) H-reflex variability extracted from data collected previously for five healthy and two participants post-stroke performing operant RF H-reflex conditioning [23], to calculate the estimated performance. Later, we normalized the estimated performance magnitude for direct comparison with real operant H-reflex conditioning performance, using the method mentioned in our previous study [23]. We compared the estimated performance from the LMM based on measured H-reflex variability with the real operant H-reflex conditioning performance.

4. The paper mentions extracting parameters from real operant conditioning data but does not provide details about the statistical methods or analysis techniques used.

>> Thank you for your question. In our method section, we have mentioned how we extracted natural biological variability of H-reflex and signal measurement noise. Also, to clarify how we extracted our biological variability, we have added additional information.

Lines 213-217:

We modeled the natural biological variability of the H-reflex (***σ^2^***) with a normal distribution obtained from a set of participants’ H-reflexes, also from previous work [23]. All analyses and distribution model fitting was performed using MATLAB software (MATLAB 2019a, MathWorks Inc., Natick, MA).

5. The authors should delve deeper into the significance of the observed relationships between feedback parameters and provide insights into how these findings can be applied or expanded upon. The manuscript should address the limitations of the study and suggest avenues for future research.

>> Thank you for your valuable suggestion. We have addressed our study’s limitations and its corresponding future research directions.

Lines 497-509:

Our simulation paradigm possesses two major limitations that preclude drawing a direct inference of how learning from simulation may be transferred to real operant H-reflex conditioning capability. First, our simulation model was designed as a one-dimensional study, which solely focused on the angle of the rotary knob with a single global maxima and minima. In the real operant H-reflex conditioning environment, however, reported operant strategy is multi-dimensional and varies within and across participants, which could lead to strategic local maxima and minima [14, 46]. Future investigations could examine learning on a trial-by-trial basis [47, 48]. Other approaches could adapt this paradigm to incorporate multi-dimensional strategies. Second, our feedback parameters (e.g., biological variability, reward threshold) were chosen based on a limited pool of 7 subjects’ data from our prior work [23]. As H-reflex is known for its large biological variability originating from various reasons [49], our two-level variability analysis can be expanded to further investigate the effect of multi-level biological variability.

6. Consider including papers using dynamic activation in regards to neural networks. This will help make the manuscript take a more round shape regarding the literature review. Try to add some relevant papers from the PLOS Journal.

>> Thank you for your valuable suggestion. We have included relevant references toward our future research direction.

Lines 503-04:

Future investigations could examine learning on a trial-by-trial basis [47, 48].

[47] Rane C, Tyagi K, Manry M. Optimizing Performance of Feedforward and Convolutional Neural Networks through Dynamic Activation Functions. Epub ahead of print 10 August 2023. DOI: 10.48550/arXiv.2308.05724.

[48] Granato G, Cartoni E, Rold FD, et al. Integrating unsupervised and reinforcement learning in human categorical perception: A computational model. PLOS ONE 2022; 17: e0267838.
