# Supplementary material for: Simulated operant reflex conditioning environment reveals effects of feedback parameters
Source: PLoS One. 2024 Mar 21;19(3):e0300338. doi: 10.1371/journal.pone.0300338 (PMC10956789; doi:10.1371/journal.pone.0300338)
Supplement: Attachment — Submitted filename: Response to Reviewers.docx [file pone.0300338.s004.docx]

We thank the editor and reviewers for their valuable comments that have strengthened the manuscript. We have carefully reviewed these comments and we believe they have been sufficiently addressed. We have primarily addressed the comments in the manuscript but have included excerpts in this document where we felt necessary. Our responses are in red font.
The line numbers refer to lines in ‘Track Changes (Simple Markup)’ mode. Thank you very much for your time, effort, and attention.

Reviewer Comments:

Reviewer 1

1. I only have a few minor recommendations before providing a decision of accept.
I am unsure if the data with your OSF directory are finalized but as I look at them in their current form they are nearly impossible to translate back to the experimental paradigm.From what I can gather, each .txt file consists of the 10 conditions (which were presented in a random sequence?) with each condition consisting of 35 trials? I cannot tell which data point corresponds to which trial (or run) or condition and I believe that the three files for each participant represents the separate sessions? But then each column within each data file has no variable name. Even as I try to carefully read this manuscript, the data in the OSF only look like random numbers. Even when I try to plot them they just look like noise. These data either need a readme file and a complete reformatting to allow outside users to understand what they are looking at. Also, the data should not just be released as individual files. There should just be one large parent file with a column for participant ID, session, and whatever strtg means in the file name.

>> Thank you for the detailed and valuable comment. We have added a readme.txt file to the osf dataset that explains the data nomenclature.

2. My motivation for this is because I wanted to look at the data as a response to my initial comment,

“In the mixed effect model did you also include trial number as a fixed effect?”

You responded that run number showed no effect on the model. This is surprising given this is a study on learning. There being no effect of run or trial number would indicate that participants do not improve overtime and thus no learning is actually taking place? When I look at fig 7, now with a y-axis label, I see now that their doesn’t appear to be any change in performance across the several runs, or at least it is impossible to infer from this graph given there is so much nested within each run (10 conditions presented at random, with 35 trials per condition). What is the explanation for this?

>> Thank you for the valuable comment. We understand the confusion because in a typical motor learning paradigm there is run-by-run learning. However, this paradigm is an unskilled task, where no useful information is transferred to the next run. For instance, the target location randomly varies by run, and the variation of strategy to reach the target is not a difficult procedure, so any learning would show a ceiling effect quickly. We did not predict any learning between runs, and did not include the statistical analysis because it would be an unnecessary test. However, as could be inferred from Figure 7, there was no learning between runs based on an LMM we ran post-hoc. We have succinctly addressed the reviewer’s concern in the limitations paragraph:

Lines 503-6:

*The current paradigm should not show run-by-run learning (Figure 7) because there was no strategy or information in this unskilled task to carry over to the following runs. However, future investigations could examine learning on a trial-by-trial basis [47, 48].*
